# Exploring the factors influencing the cloud computing adoption: a systematic study on cloud migration

**DOI:** 10.1186/s40064-015-0962-2

**Published:** 2015-04-25

**Authors:** Rashmi Rai, Gadadhar Sahoo, Shabana Mehfuz

**Affiliations:** Department of Computer Science and Engineering, Birla Institute of Technology, Mesra, Ranchi, India; Department of Electrical Engineering, Jamia Milia Islamia, Delhi, India

**Keywords:** Cloud migration, Secure migration, Legacy-to-cloud migration, Systematic literature review

## Abstract

Today, most of the organizations trust on their age old legacy applications, to support their business-critical systems. However, there are several critical concerns, as maintainability and scalability issues, associated with the legacy system. In this background, cloud services offer a more agile and cost effective platform, to support business applications and IT infrastructure. As the adoption of cloud services has been increasing recently and so has been the academic research in cloud migration. However, there is a genuine need of secondary study to further strengthen this research. The primary objective of this paper is to scientifically and systematically identify, categorize and compare the existing research work in the area of legacy to cloud migration. The paper has also endeavored to consolidate the research on Security issues, which is prime factor hindering the adoption of cloud through classifying the studies on secure cloud migration. SLR (Systematic Literature Review) of thirty selected papers, published from 2009 to 2014 was conducted to properly understand the nuances of the security framework. To categorize the selected studies, authors have proposed a conceptual model for cloud migration which has resulted in a resource base of existing solutions for cloud migration. This study concludes that cloud migration research is in seminal stage but simultaneously it is also evolving and maturing, with increasing participation from academics and industry alike. The paper also identifies the need for a secure migration model, which can fortify organization’s trust into cloud migration and facilitate necessary tool support to automate the migration process.

## 1 Introduction

Cloud computing has created a strong buzz around, both in academia and in the industry. Many SMEs (Small and Medium Enterprises) and IT companies view this technology as an opportunity for considerable business growth thereby creating competitive advantage (Buyya et al. 2009; Andrikopoulos et al. 2013). For SMEs, the central attraction for adopting cloud technology is its pay-per-use model, which delivers flexible costing options, apart from the scalability and interoperability features, which cloud environments offer. Larger enterprises are attempting to leverage this technology by considering the business continuity strategies for their exponential growth (Buyya et al. 2009; Khajeh-Hosseini et al. 2012). The traditional legacy system, which supports the core IT processes at organizations, is fraught with maintainability and scalability issues, (Khadka et al. 2013). Given the multiple benefits of cloud computing, many organizations are keen to adapt to this innovative technology. However, tackling security issues regarding the cloud and the migration process has hampered the cloud adoption rate (Rosado et al. 2012; Mather et al. 2009).

This paper is directed towards finding a viable solution to facilitate secure migration of on-premises software application to the cloud environments. Given the inherent advantages of cloud computing and the desire to migrate to cloud, there has been noteworthy research in the area of cloud migration (Khadka et al. 2013; Andrikopoulos et al. 2013). Most of the approaches have proposed frameworks, techniques, processes and methods which help in the migration and assist in decision process for migrating to cloud. For most of these approaches, the software application is in nascent stage as they are hosted on a local server, before the migration.

During the limited study, it was found that a systematic literature review of research on secure cloud migration hasn’t been undertaken. Besides considering the growing demand for migration toward cloud, there is an equal need to investigate a research framework for secure cloud migration.

A SLR identifies, classifies, and synthesizes a comparative overview of the ongoing research and enables knowledge transfer within the research community (Brereton et al. 2007). Likewise, for this paper, a SLR was conducted, with the primary objective to identify, taxonomically classify, and systematically compare the existing research, focused on planning, executing, and validating migration of legacy systems toward cloud-based software. More specifically, to the paper endeavors to answer the following questions, through conducting a methodological review of existing research:

i. What are the motivations behind migration to the cloud?
ii. What are the existing tasks, methods, and techniques to support secure migration of legacy on-premises software to cloud? In addition, what all tool support is available to achieve the objectives?
iii. What are the existing research themes? What should form future research dimensions in legacy to- cloud migration?

The objective is to systematically identify and taxonomically classify available evidence on secure cloud migration and provide a holistic comparison to analyze potential and limitations of the existing research work.

The remaining sections of this paper are structured as follows: Section 2 describes background and related research to position the contributions of this work. Section 3 explains the research methodology, research questions, and scope; Section 4 provides a reference model for state-of-the research and a characterization scheme for cloud migration; Section 5 presents the results of the systematic review; Section 6 discusses the main findings, implications, and trends followed by an analysis of its limitations in Section 7 and Section 8 concludes the paper.

## 2 Related work

The research on cloud migration is incomplete without talking about SOA (Service-Oriented Architecture. As both cloud migration and SOA exhibit numerous similarities as well as differences at the same time, it would not be appropriate to position the study on cloud migration without SOA migration. Recently, several studies have focused on migration to SOA, but not many are found for cloud migration. Both these technologies offer key benefits as reduced overall cost, business agility and easy provisioning of services to the organizations. Systematic review of 121 primary studies on SOA migration done by Khadka et al (Khadka et al. 2013) showed the use of software re-engineering reference framework for SOA migration, to give a significant view of legacy to SOA migration. This work is motivated by the research methodology used in the mentioned review work. The research agenda developed by the SEI (Software Engineering Institute) for SOA migration (Lewis et al. 2010) provides a taxonomy, which is used to classify topics into various aspects of SOA, along with cross-cutting concerns. Another survey done by Razavian & Lago (2011) with industry representatives as participants on SOA migration highlights the potential gap between the theory and practice of the SOA migration. The survey also identified future research directions in SOA migration. Work done by Pahl et al. (2013) is based on the three different case studies in industry, which proposed a common migration process, based on expert interviews. They identified a process framework for the three deployment models in cloud computing; however the work didn’t deliberate on post migration activities.

## 3 Research methodology

This research effort will thus aim to address the following Research Questions (Table 1):

**Table 1:** **Research questions and their motivation**

**Research questions**	**Motivation**
RQ1- What cloud security requirements have been addressed in recent publications (2011-2014)?	The aim is to find out what all aspects of cloud security have been researched and what all are not being researched.
RQ2- What solutions are offered to them?	The aim is to know the way/ framework through which any specific security issue has been resolved its current status.
RQ3- When did research on cloud migration become active in computing community and how it is reported?	The goal is to understand the existing gaps in cloud security framework and cloud migration process
RQ4- What are the obstacles in the cloud migration process and how it is being researched?	The objective is to probe the actual reasons for organizations not adopting cloud and the way it has been dealt in the research work.

### Methodology

**Figure 1:**
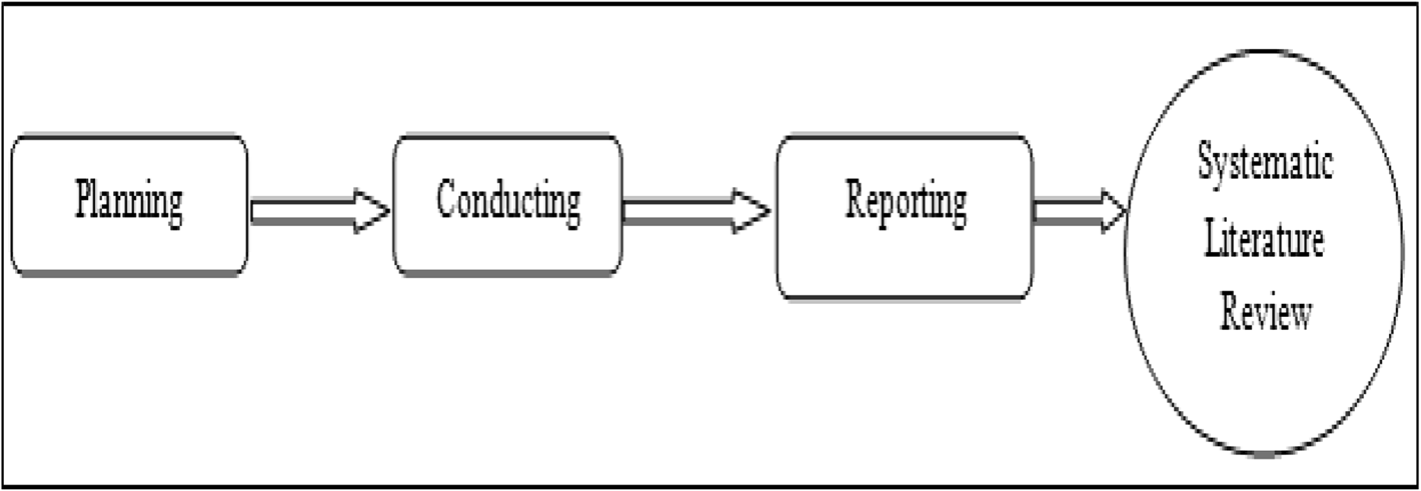
Systematic Review Process.

Systematic Literature Review (SLR) and the related guidelines (Kitchenham 2004) have been used, to answer the stated research questions. Select survey with optimal mix of participants and interactive conversation has been used to arrive at answers to some of the questions. Primary objective of systematic literature review is to provide a comprehensive summery of literature related to a research question. “*A systematic literature review is a means of identifying, evaluating and interpreting all available research relevant to a particular research question, or topic area, or phenomena of interest”*(Kitchenham 2004)*.* This kind of review involves several discrete activities. Refer to Figure 1 for the Systematic Review Process. They have been divided into three main phases, as follows:

i. Planning the review
ii. Conducting review
iii. Reporting review

### Planning the review

#### Stages involved in planning review

The following Table 2 shows the stages involved in planning the review work and the criteria chosen for the review planning.

**Table 2:** **Stages and their criteria involved in planning the review**

**Stages**	**Criteria**
Identify the need of research	- Research gap
	- Fair research
Specify research question	- Security issues
	- Migration to clouds
	- Performance and cost benefits of migration
Develop review protocol	- Research questions
	- Choosing appropriate keyword
	- Developing the search strings
	- Primary study selection

### Development of review protocol

After identifying the need of research, research questions were prepared and the review protocol was designed. Review protocol defines specific procedures for conducting the systematic review process. This procedure helps in gathering fair and unbiased information. This protocol development has different stages, such as search strategy, selection criteria, quality assessment criteria, data extraction form and data synthesis strategy.

### Search strategy

This strategy helps in answering key research questions effectively. By using keywords search strings were developed. Search strings are constructed by identifying synonyms and alternative spellings for each of the question elements and link them by using the Boolean OR and Boolean AND. Keywords in Table 3 are defined by using PICO (Population Intervention Comparison Outcomes) method (Kitchenham 2004); and are used to construct search strings. The elements of PICO is indicated below-

i. **Population**: The population might be any of the specific role, application and area.
  Population- Cloud Computing
ii. **Intervention**: The intervention is the tool or technology or procedure that addresses a specific issue.
  Intervention-Cloud Migration
iii. **Comparison**: This is a tool or technology or procedure with which intervention is being compared.
  Comparison- Legacy on premises application
iv. **Outcomes**: Outcomes should relate to factors of importance to practitioners such as improved security, reliability and cost benefits. All outcomes should be specified.
  Outcomes - Secure framework for migration, improved security aspects, performance, cost benefits, applications, tools and techniques.

**Table 3:** **Research questions and keywords**

**S.No.**	**Research questions**	**Keywords**
RQ1	Why is there an urge to migrate to cloud?	Need, benefits, requirement, motivation, cloud, migration
RQ2	What are the challenges in cloud migration process?	Challenges, issues, process, migration, cloud
RQ3	What are the existing processes or tools for cloud migration?	Tools, process, standards, framework
RQ4	What is the current state and ongoing research issues for secure cloud migration?	Current state, research issues, cloud migration, secure migration

### Search string

Following search strings in Table 4 are appropriately designed by using keywords, which are derived from research questions through PICO method. These search strings are constructed by using Boolean ANDs and ORs.

**Table 4:** **Research questions and search strings**

**Research questions**	**Search strings**
RQ1	(Advantages OR Need OR Urge OR Benefits) AND (Cloud Computing OR Cloud Migration OR Migration OR Adoption)
RQ2	(Challenges OR Issues OR Security Concerns) AND (cloud computing OR Cloud Migration OR Migration Process OR Migration) AND (Security OR issues OR problems OR Threats)
RQ3	(Cloud Computing OR Cloud Migration OR Migration) AND (Tools OR Process OR Framework OR Standards OR Benchmark)
RQ4	(Cloud Computing OR Cloud Migration OR Migration) AND (Current state OR SLR OR Systematic Literature Review) AND (Secure Cloud OR Secure Cloud Migration)

### Resources

Search strings are used in digital libraries for getting related research content. The articles, journals, conference papers, and workshop papers have been identified from the most authentic digital databases, that are scientifically and technically peer reviewed. Some of the databases are as follows -

i. ACM Digital Library
ii. Springer Link
iii. Science Direct
iv. IEEE Xplore
v. Google Scholar
vi. Wiley
vii. Compendex
viii. Reports and white papers published by groups and organizations working on cloud computing (e.g. CSA, NIST, ENISA etc.)

### Inclusion criteria

The following inclusion criteria (Table 5) were used to include the selected papers.

**Table 5:** **Inclusion criteria**

**S.No.**	**Stage**	**Criteria**
1.	Over All	- Language: English
		- Paper published in journal/conference/workshop/ web articles.
		- Date of publish
		- Non-duplicate
2.	Title, Keywords, Abstract	- Based on keywords and search strings.
		- Based on the content, which matches to the research questions.
3.	Introduction & Conclusion	- Contains cloud security and migration aspects.
		- Primarily focuses on answering the research questions.
4.	Full Text	- Presence of the empirical data in the paper.
		- Key focus on security, migration, benefits, challenges and tools in cloud.

### Exclusion criteria

The research articles were excluded that didn’t meet the criteria mentioned as indicated above in Table 5 and the following parameters:

i. Articles shorter than 6 pages
ii. Editorials and Abstracts
iii. No-peer reviewed studies

### Survey on secure migration process

Survey on the secure migration process was done, to identify key concerns related to the secure adoption of cloud by both industry and academia and also to seek their expert opinion on the proposed framework. All the participants had considerable understanding of cloud computing, its multiple offerings, related technologies, and many hands-on expertise to Cloud environment. As part of their work, the participants were part of the team, which migrated different types of applications to Cloud (including Amazon EC2, Amazon RDS, S3, Simple DB, Windows Azure etc.). With their exposure to the cloud computing environment, they were reliable and valuable participants for the discussion.

### Participants

**Figure 2:**
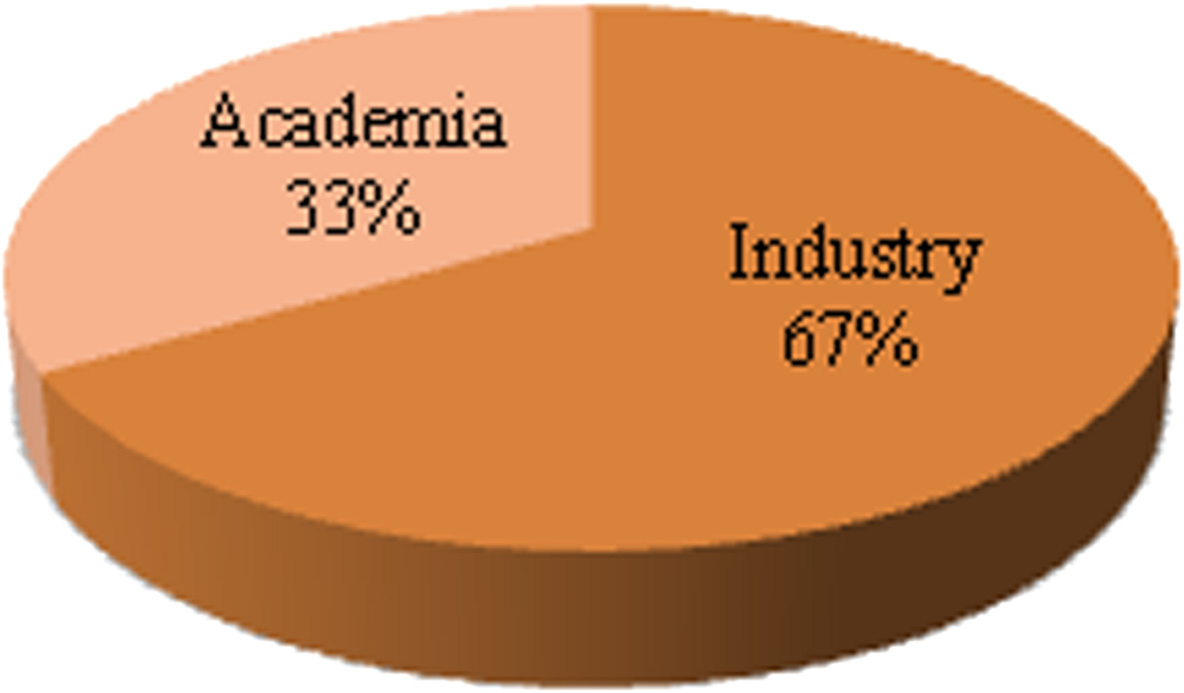
Sample Characteristics (Total = 9).

The discussion and the survey were carried out with 9 participants from industry and academia individually. Refer Table 6 for the survey participants. The sample characteristics are shown in Figure 2.

**Table 6:** **Survey participants**

**Field**	**Participants**	**Nos.**
Industry	Software Engineers, with 10 years’ experience in software development and 3 years’ experience with Cloud computing	3
	Entrepreneurs cum cloud experts with their own startup firms providing cloud migration services.	2
	Cloud Architect with 9 years of experience in software development and 4 years of experience in cloud design and migration	1
Academics	Senior Professor with 20 years of Global experience in teaching and guiding students in the Computer Science.	1
	Ph.D. Research Students, who are in their final stage of thesis and their Ph. D. topics are related to Cloud Computing performance.	2
Total		9

### Discussion protocol

Each participant was asked similar questions in three steps:

i. Firstly, each participant was asked for his opinions on the state of art of cloud computing, existing security concerns and the taxonomy of migration tasks. The participants were encouraged to suggest adding more tasks, removing some, or re-categorizing a task.
ii. Secondly, the framework for secure cloud migration was presented to the participants, and they were asked for their expert opinion and advice on the model.
iii. Thirdly, each participant was asked to describe a cloud migration project, which they worked on, together with the time spent on each migration task in that project.

The discussion was completed with each participant individually, without knowledge of other participants’ answers in the first round. Second round of discussions was conducted with each participant again, but this time with knowledge of other participants’ replies, to decrease the range of answers. This is known as Delphi technique and its helps combine experts’ opinion for a better judgment (Shepperd & Schofield 1997). Interactive conversation survey method was used for conducting fair survey. Here in this method, professional websites as m LinkedIn, different blogs related to Cloud and Cloud Migration was leveraged for conducting the survey. Questionnaires were posted into those sites to have an interactive conversation with the participants, regarding cloud. Author also had few conversations with select organizations using live chat who are working in the field of Cloud.

## 4 A 5-phase model for classification and comparison of cloud migration research

**Figure 3:**
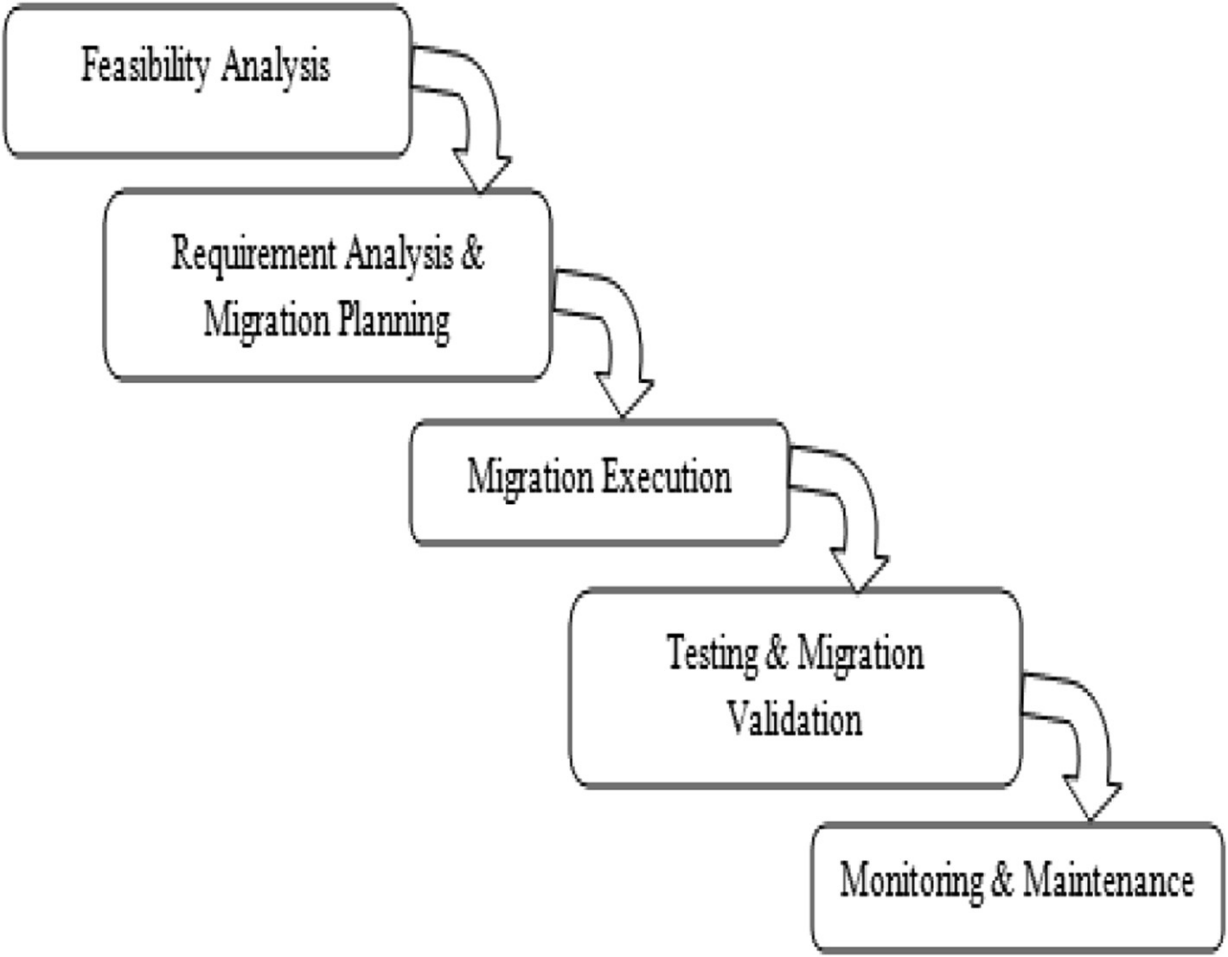
5-Phase Cloud Migration Model.

**Figure 4:**
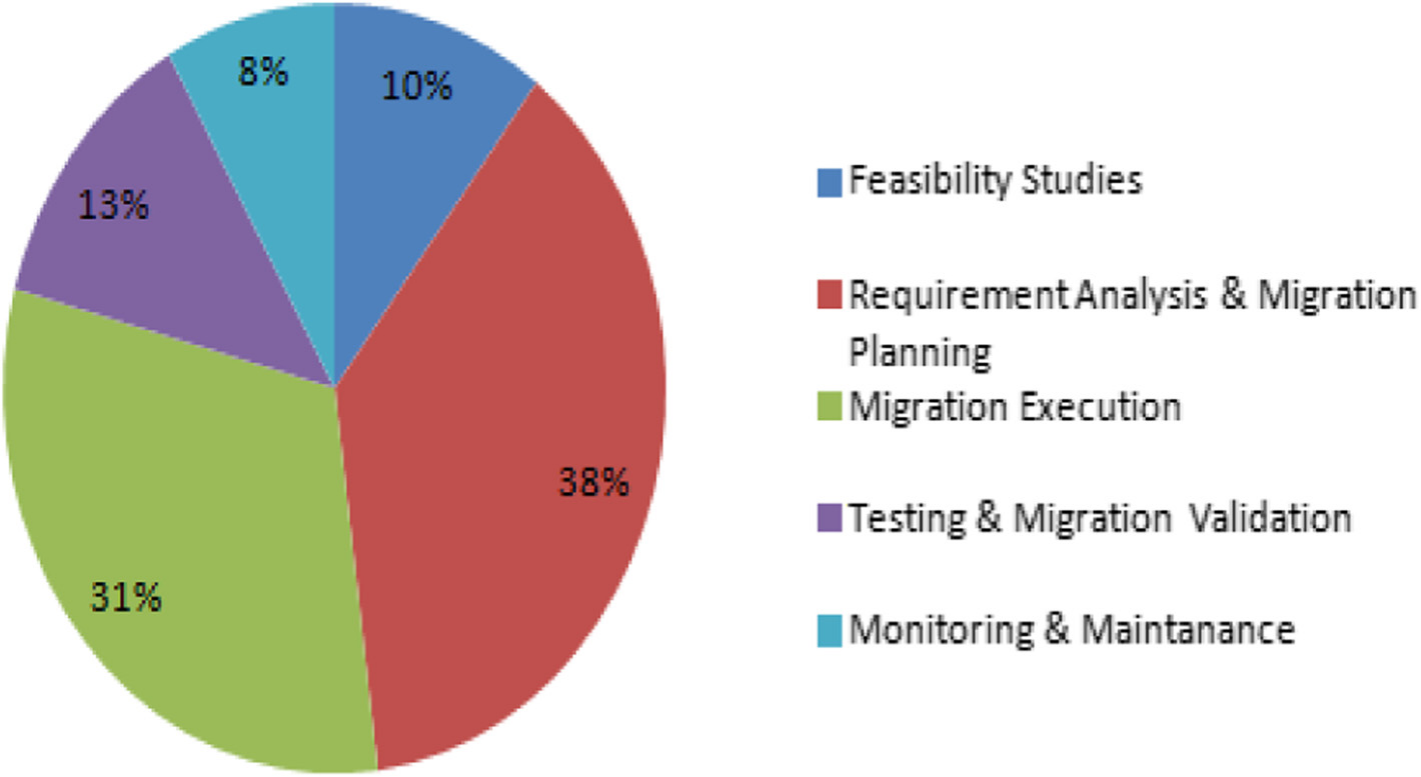
Distribution of studies according to 5-Phase Cloud Migration Model.

In this section, a conceptual model called as ‘5-Phased Cloud Migration Model’ has been introduced, to classify and categorize cloud migration research, in terms of distinct phases or processes involved in the cloud migration. While developing this reference model, situational method engineering has been adopted to consolidate the existing frameworks (e.g.P3, P4, P9, P23 etc.) in cloud migration. Method engineering follows a bottom-up approach by identifying low level activities and techniques. These low level activities are then categorized to form generalized processes and phases. Alternatively, a top-down approach forms a framework or a conceptual model consisting of phases, processes and activities. Based on these existing frameworks and guidelines, we have identified the key phases in cloud migration. By reviewing the primary studies and exploring the defined migration tasks, migration process has been categorized in five phases. Figure 3 below represents the ‘5-phased cloud migration model’ which is also inspired by the well-known ‘Water Fall model from the Software Development Life Cycle (SDLC). The proposed conceptual model consists of five phases. Figure 4 shows the distribution of studies according to 5-Phase Cloud Migration Model

### Phase-1: Feasibility study [5 studies]

In the first phase the goal is to identify or determine whether the cloud migration is financially/ technically feasible or not [P2] [P6] [P8] [P13] [P14].

### Phase-2: Requirement analysis & migration planning [18 Studies]

In this phase, a detailed assessment of the existing IT environment is done. The objective is to understand the applications that are appropriate for moving into the Cloud [P2] [P15] [P16] [P18], decision making regarding which cloud provider to choose [P3] [P5] [P6] [P9] [P13] [P16], which part of the application to be migrated [P3] [P6] [P13] [P15] and which services to use [P4] [P6] [P11] [P12] are conducted. The output of this phase is a detailed migration plan document.

### Phase-3: Migration execution [15 Studies]

In the migration execution phase, the actual migration of data and application is carried out. The process like data extraction [P2] [P11] [P22], code modification [P15] [P17] [P20], architecture recovery [P9] [P11] [P2] [P22] [P18] [P20], cloud migration [P9] [P22] [P17] etc. are actually implemented.

### Phase-4: Testing & migration validation [6 Studies]

In the fourth phase, testing and evaluation is done to validate the migrated system [P9] [P11] [P15] [P22] [P17] [P18].

### Phase-5: Monitoring & maintenance [4 Studies]

The last and fifth phase is required to maintain and monitor the migrated systems. Not much evidence could be found for this activity in the selected study except for some related activities, as governance [P2] and training [P11] [P21] [P17].

## 5 Results

In this section we have discussed the results of the SLR process based on the research questions that were defined in Section 1.

### Key factors for migration

Based on the existing literature, the research question (RQ1) has been answered and the key reasons for adoption of clouds have been identified. Some of the key drivers for the adoption of clouds are:

i. Cost saving
ii. Optimum resource utilization
iii. Unlimited scalability of resources
iv. Less maintainability

These key drivers for cloud adoption have been identified from the selected primary studies and have been presented in a tabular form along with the author’s name and title. For instance the cost saving has been the major driver for cloud adoption as mentioned and discussed about in several studies, also indicated in the Table 7 below.

**Table 7:** **Key factors for migration**

**S.No.**	**Key factors**	**Authors/Year/Title**
1	**Cost saving** P[5,11,8,13,14,16,17,23]	- Beserra et al. (2012) **Cloudstep: A Step-by-Step Decision Process to Support Legacy Application Migration to the Cloud**
		- Mohagheghi *et al* (2011) **Software Engineering Challenges for Migration to the Service Cloud Paradigm**
		- Khajeh-Hosseini et al. (2012) **The Cloud Adoption Toolkit: supporting cloud adoption decisions in the enterprise**
		- Tak et al. (2011) **To Move or Not to Move: The Economics of Cloud Computing**
		- Yam et al (2011) **Migration to Cloud as Real Option Investment decision under uncertainty**
		- Zardari et al (2011) **Cloud Adoption: A Goal-Oriented Requirements Engineering Approach**
		- Tran et al. (2011a) **Application Migration to Cloud: A Taxonomy of Critical Factors**
		- Hajjat et al (2010) **Cloudward Bound: Planning for Beneficial Migration of Enterprise Applications to the Cloud**
2	**Efficient resource utilization** P[10,5,12,15,23]	- Frey et al (2011) **An Extensible Architecture for Detecting Violations of a Cloud Environment’s Constraints During Legacy Software System**
		- Beserra et al. (2012) **Cloudstep: A Step-by-Step Decision Process to Support Legacy Application Migration to the Cloud**
		- Lloyd et al (2011) **Migration of Multi-tier Applications to Infrastructure-as-a-**
		- **Service Clouds: An Investigation Using Kernel-based Virtual Machines**
		- Chauhan et al (2011) **Migrating Service-Oriented System to Cloud Computing: An Experience Report**
		- Hajjat et al (2010) **Cloudward Bound: Planning for Beneficial Migration of Enterprise Applications to the Cloud**
3	**Unlimited scalability of resources** P[5,15,12,11,13,16]	- Baserra et al (2012) **Cloudstep: A Step-by-Step Decision Process to Support Legacy Application Migration to the Cloud**
		- Chauhan et al (2011) **Migrating Service-Oriented System to Cloud Computing: An Experience Report**
		- Lloyd et al (2011) **Migration of Multi-tier Applications to Infrastructure-as-a-Service Clouds: An Investigation Using Kernel-based Virtual Machines**
		- Mohagheghi *et al* (2011) **Software Engineering Challenges for Migration to the Service Cloud Paradigm**
		- Tak et al (2011) **To Move or Not to Move: The Economics of Cloud Computing**
		- Zardari et al (2011) **Cloud Adoption: A Goal-Oriented Requirements Engineering Approach**
4	**Less maintainability** P[23,8,14]	- Hajjat et al (2010) **Cloudward Bound: Planning for Beneficial Migration of Enterprise Applications to the Cloud**
		- Khajeh-Hosseini et al. (2012) **The Cloud Adoption Toolkit: supporting cloud adoption decisions in the enterprise**
		- Yam et al (2011) **Migration to Cloud as Real Option Investment decision under uncertainty**

### Challenges in cloud migration process

In our previous work (Rashmi et al. 2013) we have identified (refer Table 8) various challenges in the cloud migration process and have attempted to answer the RQ2 by listing out various challenges which organizations face, while adopting the cloud.

**Table 8:** **Migration challenges**

**Migration challenges**	**Description**
Business factors	→Costs
	→Existing investments in IT
	→Data security
	→Regulations
	→Provisioning
Technical factors	→Existing infrastructure
	→Security architecture
	→Complexity
	→Network and support
	→IT skills
	→Service Level Agreements (SLAs)

### Existing processes or frameworks for secure cloud migration

To answer RQ3, classification of different migration types given in (Khajeh-Hosseini et al. 2012) has been referred to. This work considers different application layers and different degree of adaptation required to enable migration. It classifies the migration process into following types:

Type 1: Replace
  This type of migration replaces one or more legacy component with cloud services. This is least invasive of all types and requires data or business tiers to be migrated to the cloud stack. This type of migration is done by reconfiguring the components and is done to adjust incompatibilities, to use functionalities of the migrating layer. Replace type of migration couldn’t be identified in the selected studies. This particular type is not very popular as much as pure cloud enabler and hence the evidence in probably not available.
Type 2: Partially Migrate
  This one partially migrates some of the systems components to the cloud. There are quite few papers on the partial migration where the organizations have migrated one or more application layer implementing a particular functionality in the cloud.
Type 3: Migrate the whole application
  This is a perfect example of migration where the whole application is encapsulated in one or more virtual machines, which are already running into the clouds. This one also doesn’t need many changes to the application, assuming the application can be ported ‘as is’ into a virtual machine.
Type 4: Cloudify
  Cloudify is an example of full migration, where an application is converted to a fully-fledged cloud enabled system by composing cloud services.

Table 9 below categorizes all the four types of migration along with the Cloud Deployment models, which were used in the migration process. The table also identifies various tools/frameworks which are used in the selected studies.

**Table 9:** **Categorization of primary studies based on migration type, deployment model and tool support**

**ID**	**Author/Year/Title**	**Migration Type**	**Cloud Deployment Model**	**Framework/Tools Support**
P1	Frey et al. (2013a) **Search-Based Genetic Optimization for Deployment and Reconfiguration of Software in the Cloud**	Cloudify	IaaS	CDOXplorer
P2	Andrikopaulas et al (2013) **How to Adapt Applications for the Cloud Environment: Challenges and Solutions in Migrating Applications to the Cloud**	Cloudify	SaaS,Iaas,PaaS	…
P3	Fittakau et al (2012)**CDOSim: Simulating Cloud Deployment Options for Software Migration Support**	Cloudify	IaaS	CDOSim
P4	Menzal et al (2012) **CloudGenius: Decision Support for Web Server Cloud Migration**	Migrate the whole stack	PaaS,IaaS	CloudGenius
P5	Baserra et al (2012) **Cloudstep: A Step-by-Step Decision Process to Support Legacy Application Migration to the Cloud**	Migrate the whole stack	NM	…
P6	Vu and Asal (2012) **Legacy Application Migration to the Cloud: Practicability and Methodology**	Partially Migrate	PaaS,IaaS	…
P7	Frey et al. (2013b) **Automatic conformance checking for migrating software systems to cloud infrastructures and platforms**	Cloudify	PaaS,IaaS	CloudMIG Xpress
P8	Khajeh-Hosseini (2012) **The Cloud Adoption Toolkit: supporting cloud adoption decisions in the enterprise**	Partially Migrate	NM	Cloud Adoption Toolkit
P9	Frey et al (2011) **The CloudMIG Approach: Model-Based Migration of Software Systems to Cloud-Optimized Applications**	Cloudify	PaaS,IaaS	CloudMIG
P10	Frey et al (2011) **An Extensible Architecture for Detecting Violations of a Cloud Environment’s Constraints During Legacy Software System Migration**	Cloudify	IaaS	CloudMIG Xpress
P11	Mohagheghi *et al* (2011) **Software Engineering Challenges for Migration to the Service Cloud Paradigm**	Migrate the whole Stack	NM	…
P12	Lloyd et al (2011) **Migration of Multi-tier Applications to Infrastructure-as-a-Service Clouds: An Investigation Using Kernel-based Virtual Machines**	Cloudify	IaaS	Eucalyptus
P13	Tak et al (2011) **To Move or Not to Move: The Economics of Cloud Computing**	Cloudify	IaaS,SaaS	…
P14	Yam et al (2011) **Migration to Cloud as Real Option Investment decision under uncertainty**	Partially Migrate	NM	…
P15	Chauhan et al (2011) **Migrating Service-Oriented System to Cloud Computing: An ** **Experience Report**	Cloudify	SaaS,PaaS. IaaS	…
P16	Zardari et al (2011) **Cloud Adoption: A Goal-Oriented Requirements Engineering Approach**	Partially Migrate	NM	…
P17	Tran et al (2011) **Application Migration to Cloud: A Taxonomy of Critical Factors**	Partially Migrate	NM	…
P18	Babar and Chauhan (2011) **A Tale of Migration to Cloud Computing for Sharing Experiences and Observations**	Migrate the whole stack	SaaS,IaaS	…
P19	Tran et al (2011) **Size Estimation of Cloud Migration Projects with Cloud Migration Point (CMP)**	Partially Migrate	PaaS,IaaS	…
P20	Yu. et al (2011) **A Practical Architecture of Cloudification of Legacy Applications**	Cloudify	SaaS,Iaas,PaaS	…
P21	Khajeh-Hosseini et al. (2010) **Cloud Migration: A Case Study of Migrating an Enterprise IT System to IaaS**	Migrate the whole stack	NM	…
P22	Ward et al (2010) **Workload Migration into Clouds – Challenges, Experiences, Opportunities**	Partially Migrate	NM	Darwin
P23	Hajjat et al (2010) **Cloudward Bound: Planning for Beneficial Migration of Enterprise Applications to the Cloud**	Partially Migrate	PaaS,IaaS	Cloudward Bound

### Current state and ongoing research issues in secure cloud migration

In this section, we have attempted to answer RQ 4 by carrying out a systematic review of the existing approaches for legacy to cloud migration. This review is done to summarize the existing approaches, models, tools and techniques and also to identify and analyze the security issues considered in these migration approaches. The focal objective is to identify the possible solutions offered to address the security concerns or needs in the cloud migration process. In this regard, a set of approaches have been collated which is pertinent for this analysis. The details are as summarized in the Table 10.

**Table 10:** **Primary studies on secure cloud migration and key findings**

**ID**	**Author /Year**	**Key Findings**
P24	Frey and Hasselbring (2010)	• Still in nascent stage
		• Migration to Paas & IaaS
		• Six step migration process
		• None of the security aspects discussed
P25	Zhang et al. (2009)	• A case study for a scientific software migration
		• Migration considers only SaaS
		• Talk about security in a general non-specific way
P26	Parastoo et al. (2010)	• Tools for model driven migration
		• No specific security aspect covered
P27	Hu and Klein (2009)	• Case study for migrating in oil and gas industry
		• Cost analysis and decision support tool
P28	Khajeh-Hosseini et al. (2010a, 2010b)	• Tools to support decision making during migration
		• Talks about the risks and benefits
P29	Khajeh-Hosseini et al. (2011)	• Considers security as a challenge
		• Doesn’t deliberate on how the data will be migrated
P30	Kaisler and Money (2011)	• Service migration in cloud computing
		• Examines security and integration issues
P31	Kalloniatis et al.(2013)	• Critical threats in cloud computing identified
		• Security and privacy oriented concepts analyzed

## Conclusion

The central objective of this review paper was to consolidate the existing research on cloud migration and identify the security concerns reflected in these selected review papers. The foremost contribution of this systematic review is the proposition of conceptual model for cloud migration for the characterization of the studies and a comparative analysis of the existing literature through the model, to indicate the tools and techniques used in the various studies. Authors have also tried to identify the security concerns in the existing literature studies on cloud migration. Authors have defined the cloud migration process in a 5-Phased model. The five phases are as follows-

i. Feasibility study
ii. Requirement analysis & migration planning
iii. Migration execution
iv. Testing & migration validation
v. Monitoring & maintenance

After analyzing the studies collected through this Systematic Review Process, a number of research challenges were observed and which indicated future directions of this research.

i. *Growing maturity of cloud migration* – Even though it has been acknowledged that the maturity of the cloud migration is in its pivotal stage, one can observe a clear sign of growth by observing various types of cloud migration being reported in the literature (already discussed in Section 2.3). Proper validation across all these types of migration is an area that needs immediate attention by the cloud researchers.
ii. *Need for more results on cloud migration evaluation* - By observing the results on cloud migration in the selected studies one can clearly identify the need for more and more results and real-time case studies from industries on cloud migration. More evaluation, survey and experience reports on legacy-to-cloud migration will be needed, which will result in more trust and confidence of researchers regarding the validity of cloud migration research.
iii. *Need of a comprehensive migration framework –* Although, the Authors have presented a 5-Phase model for cloud migration in Section 4, the cloud researchers needs to propose a more comprehensive framework such as the ones proposed for SOA migration (Discussed in Section 2) with tangible evidence of solutions in terms of methods and techniques.
iv. *Solutions to address Security Concerns –* As per the distribution of studies based on the 5-Phase model for cloud migration (Figure 4), the main focus of the research is on the requirement analysis and cloud migration planning (approx. 38%), however very few of them address the security concerns hovering over the cloud migration (discussed in Section 5.4).

To summarize, one can conclude that cloud migration is still in its nascent stage, but is maturing at a fast pace. The Authors have acknowledged the call for a tangible secure migration framework, to facilitate systematic and trustworthy migration to the cloud.
